# Improving Mechanical Properties of Co-Cr-Fe-Ni High Entropy Alloy via C and Mo Microalloying

**DOI:** 10.3390/ma17020529

**Published:** 2024-01-22

**Authors:** Yukun Lv, Yangyang Guo, Jie Zhang, Yutian Lei, Pingtao Song, Jian Chen

**Affiliations:** School of Materials Science and Chemical Engineering, Xi’an Technological University, Xi’an 710021, China

**Keywords:** microstructure, microalloying, HEA, strengthening

## Abstract

The as-cast [Co_40_Cr_25_(FeNi)_35−y_Mo_y_]_100−x_C_x_ (x = 0, 0.5, y = 3, 4, 5 at.%) HEAs (high-entropy alloys) were prepared by a vacuum arc melting furnace and were then hot rolled. The effect of C and Mo elements on the microstructure evolution and mechanical properties of HEAs was systematically analyzed. The results showed that when no C atoms were added, the HEAs consisted of FCC + HCP dual-phase structure. In addition, as the Mo content increased, the grain size of the alloy increased from 17 μm to 47 μm. However, only the FCC phase appeared after adding 0.5 at.% carbon in Mo microalloyed HEAs, and the grain size of the Mo_4_C_0.5_ HEA decreased significantly. Due to the Mo atom content exceeding the solid solution limit, the carbides of Mo combined with the C element appeared in the Mo_5_C_0.5_ HEA. The strength of C and Mo microalloyed HEAs significantly increased compared to HEAs with no C added. However, the Mo_4_C_0.5_ HEA exhibited excellent comprehensive mechanical properties, which was superior to a majority of reported HEAs and conventional metal alloys. Its yield strength, tensile strength, and elongation were 757 MPa, 1186 MPa, and 69%, respectively. The strengthening mechanism was a combination of fine grain strengthening, TWIP effect, and solid solution strengthening.

## 1. Introduction

Unlike conventional alloys that are dominated by a single element, high-entropy alloys (HEAs) are composed of a mixture of five or more elements [1]. Due to their unique design concept different from traditional alloys, their synthetic mechanical properties are excellent [2,3,4,5] and mainly exhibit high hardness, high ductility, and good thermal stability, wear resistance, and corrosion resistance.

Although strength and plasticity are a trade-off relationship [6], grain refinement [7], micro-strip-induced plasticity (MBIP), twin-induced plasticity (TWIP), and transformation-induced plasticity (TRIP) are important deformation mechanisms used to strengthen and toughen materials to conquer the trade-offs. HEAs with low stacking fault energy (SFE) (~25 MJ/m^2^) can promote twinning, and the TWIP effect greatly promotes the plasticity of the alloy [8]. The TWIP effect is widely used in high-strength alloys, which exhibit a good combination of strength and ductility. For example, due to the generation of nano twins in the CoCrFeMnNi HEAs that increases the resistance to dislocation movement and improves the work hardening ability, the alloys have excellent tensile properties [9]. Gludovatz et al. [10] reported that NiCoCr medium entropy alloy has a tensile strength of 1 GPa at room temperature, while still having 70% elongation after fracture. Its deformation mode was dominated by the TWIP effect, which provided a high work hardening rate during the deformation process. The TRIP effect refers to the martensitic transformation under deformation. Martensitic Flat acts as a channel for blocking the dislocation slip, where dislocations accumulate and produce back stress at the interface with the matrix, thus blocking the movement of other dislocations and improving the work hardening rate of the alloys. The low-temperature and high-pressure torsion experiments conducted with CoCrFeMnNi high entropy alloys were carried out by Shahmir et al. [11], resulting in FCC phase transfer to HCP and BCC phases and significant grain refinement during deformation. This was due to a decrease in stacking fault energy at low temperatures. Li [12] successfully prepared Fe_50_Mn_30_Co_10_Cr_10_ TRIP HEA with the highest product of strength (330 MPa) and elongation (~73%). Ulteriorly, Li [13] also induced the principle of TWIP and interstitial TWIP-HEA and mixed them into HEAs. This phenomenon was also reported by Praveen et al. [14] in the medium entropy alloy (MEA) CoCrNi with a higher tensile strength and fracture elongation. 

HEAs with unique microstructures also exhibited a balanced strength–ductility duo to the addition of metallic or non-metallic elements, which significantly enhanced the mechanical properties of equal or near-equimolar HEAs [15,16,17]. In the new study, it was shown that the interstitial carbon atoms were dissolved into the Fe_50_Mn_30_Co_10_Cr_10_ HEA, and the solution strengthening and TWIP effect occurred during plastic deformation. Li [18] added 0.5 at.% C to the Fe_50_Mn_30_Cr_10_Co_10_ HEA. It was found that the SFE of the matrix increased due to the interstitial strengthening of carbon. The Fe_40.4_Ni_11.3_Mn_34.8_A_l7.5_Cr_6_ HEA with carbon atoms was investigated by Wang et al. [19], in which the yield strength and ductility were greatly improved when carbon atom content was added to 1.1 at.%. Cheng et al. [20] added 0.5 at% C to FeCoCrNiMn HEA, and the results showed that carbon had higher twinning activity and higher strength effect than that without carbon. The introduction of carbon atoms into HEAs can reduce the SFE effectively and change the mode of dislocation slip during plastic deformation. Additionally, carbon atoms increase the work hardening rate and delay the necking phenomenon. Guo et al. [21] prepared (FeCoCrNiMn)_100−x_C_x_ high-entropy alloys with different carbon contents, and after cold rolling and annealing processes, it was found that there were fine M_23_C_6_ carbides in the high entropy. This carbide precipitate phase inhibited the migration of grain boundaries and played a strong pinning role, ultimately resulting in an increase in strength due to the effects of fine-grain strengthening and precipitation strengthening.

Mo is a particularly effective microalloying element, since it is not only dissolved into the FCC solid solution but also readily forms the hard intermetallic compound [22,23,24]. Mo has a larger atomic radius and is mainly used for displacement solid solution strengthening. The microstructure, mechanical, and corrosion properties of the single-phase and the eutectic high-entropy alloy by Mo addition have been extensively studied [25,26,27]. For example, Bae et al. [25] investigated the effects of Mo content on the microstructural and mechanical properties of Co-Cr-Fe-Ni-Mo alloys. The results demonstrated that the precipitation of the hard intermetallic μ phase increased with increasing Mo content, and the formation of the μ phase decreased the rate of recrystallization and grain growth. Wei et al. [28] found that the addition of Mo atoms increased the strength of the Co-Cr-Fe-Ni HEAs. Adding 5% Mo atoms to the alloy can increase its plastic elongation to 96%. Li et al. [29] aimed to improve the solid solubility of Mo atoms in the equiatomic ratio CoCrFeNi HEA; the Cr element was easily formed in the μ phase with an Mo element. However, the Ni_1.8_Co_0.95_Cr_0.8_Fe_0.25_Mo_0.475_ high-entropy alloy had a high degree of lattice distortion and solid solution strengthening effect, while the alloy still maintained a single-phase FCC structure without precipitation.

An et al. [30] used a powder metallurgy method to prepare a series of (CoCrFeNiAl_0.5_)_1−x_(MoC)_x_ high-entropy alloys containing C atoms and Mo atoms and then obtained fine equiaxed grains through hot extrusion. When adding 8 at.% MoC, the tensile strength of the alloy increased to 1280 MPa, while still maintaining a 7% elongation. The increase in strength was attributed to solid solution strengthening, precipitation strengthening, and fine grain strengthening. However, there were few reports on adding C and Mo to Co-Cr-Fe-Ni HEAs simultaneously. The addition of appropriate amounts of Mo and C can change the deformation mechanism of HEAs, ultimately improving comprehensive mechanical properties. The microstructure evolution and mechanical properties of these alloys deserve further research. This work addresses this issue by evaluating the microstructural evolution and mechanical properties of a series of [Co_40_Cr_25_(FeNi)_35−y_Mo_y_]_100−x_C_x_ (x = 0, 0.5, y = 3, 4, 5 at.%) HEAs.

## 2. Materials and Methods

**Figure 1:**
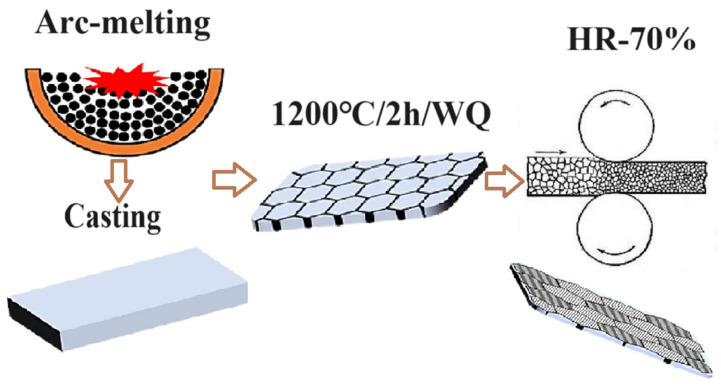
Technological process of HEAs.

The [Co_40_Cr_25_(FeNi)_35−y_Moy]_100−x_C_x_ (x = 0, 0.5, y = 3, 4, 5 at.%) HEA ingots were prepared by arc-melting pure elements (≥99.9 wt.%) under a Ti-gettered high-purity argon atmosphere in a water-cooled Cu crucible (see Figure 1). For convenience, the [Co_40_Cr_25_(FeNi)_35−y_Moy]_100−x_C_x_ (x = 0, 0.5, y = 3, 4, 5 at.%) samples are referred as Mo_3_, Mo_4_, Mo_5_, Mo_3_C_0.5_, Mo_4_C_0.5_, and Mo_5_C_0.5_, respectively (see Table 1). The ingots were melted five times to achieve a good homogeneity. The as-casted HEA was solution treated at 1200 °C for 2 h and then water quenched to obtain a complete uniform microstructure. About 70% hot rolling reduction was carried out. Flat tensile testing specimens with a gage geometry of 22 × 2.5 × 1.5 mm^3^ were cut along the rolling direction (RD) of the plate. Uniaxial tensile tests were conducted at room temperature with a universal testing machine (UTM5105 electronic universal testing machine) at a strain rate of 1 × 10^−3^ s^−1^. The tensile tests were repeated for three samples to confirm reproducibility. A Lab XRD-6000 X-ray diffraction (XRD), with a scanning rate of 0.2°/min from 20° to 100° under 40 kV and 40 mA, was adopted to determine the crystal structure. The microstructure was characterized by an FEI Quanta-400F scanning electron microscope (SEM). Electron backscattered diffraction (EBSD) analysis was performed at 20 kV with a working distance of 18 mm and a tilt angle of 70°. The nalysis software (HKLCHANNEL 5 version 5.0.9.0) was utilized to interpret the EBSD data.

## 3. Results and Discussion

**Figure 2:**
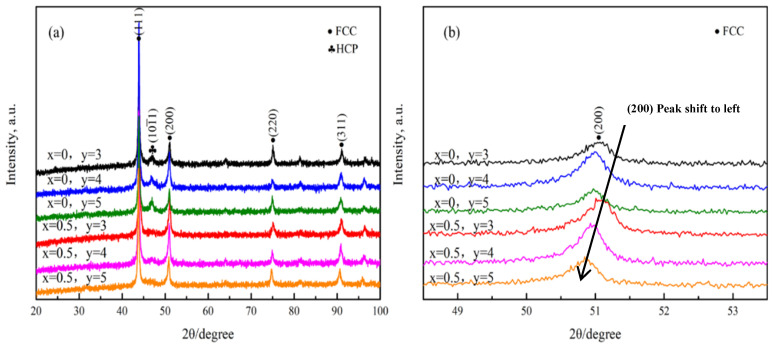
(**a**) XRD patterns of [Co_40_Cr_25_(FeNi)_35y_Mo_y_]100_x_C_x_ (x = 0, 0.5, y = 3, 4, 5 at.%) HEAs; (**b**) XRD patterns of local enlarged.

**Figure 3:**
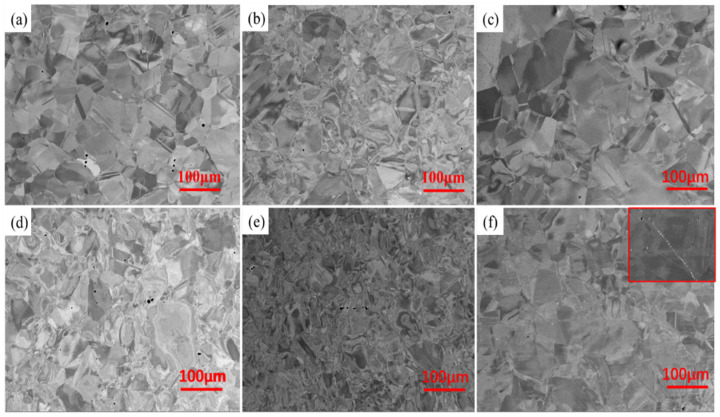
SEM-BSE image of HEAs: (**a**) Mo_3_, (**b**) Mo_4_, (**c**) Mo_5_, (**d**) Mo_3_C_0.5_, (**e**) Mo_4_C_0.5_, and (**f**) Mo_5_C_0.5_.

**Figure 4:**
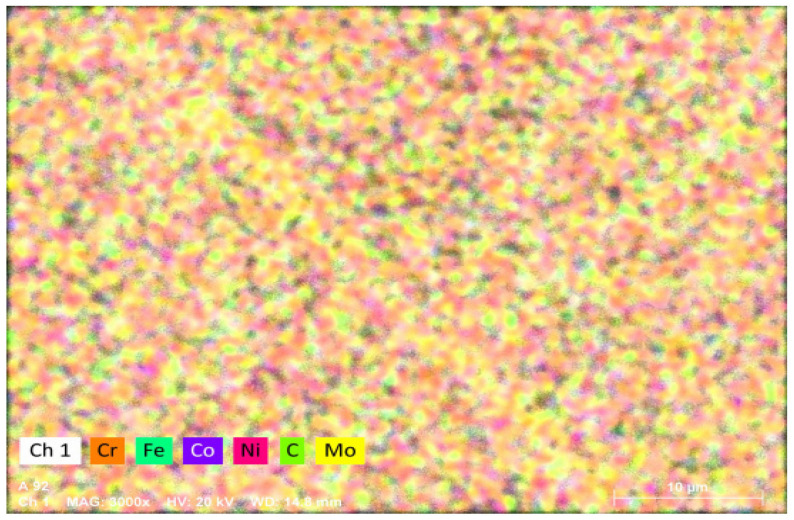
EDS of the hot-rolled Mo_5_C_0.5_ HEA.

The XRD diffraction patterns of the HEAs are shown in Figure 2a. When C atoms were not added (x = 0), the diffraction peak of the sample corresponded to the FCC + HCP dual phase in Mo_3_, Mo_4_, and Mo_5_ HEAs. However, after the addition of carbon atoms (x = 0.5), the reflection of the alloy corresponding to the HCP phase disappeared, and only the FCC diffraction peak at the 2θ value of ~43.4° was observed. It is meaningful that HEAs could maintain a uniform solid solution phase composition when a small amount of carbon was added. Figure 2b shows more detailed information on the FCC phase (200) peak. From the graph, it can be seen that, both before and after the addition of C, the (200) peak shifted to the left significantly with the increase of Mo and C atoms, indicating that the increase of Mo and C atoms could increase the lattice distortion degree of the HEAs. Mo in solid solutions can expand the lattice parameter, and the solid solution C atoms further increase the lattice distortion degree. The SEM-BSE images of the hot-rolled HEAs are shown in Figure 3. The grain size was uniform, and no component segregation and dendrites appeared. It was observed that a large number of annealing twins appeared in some deformed grains. This was mainly attributed to the not fully dynamic recrystallization process during hot rolling. The energy spectrum analysis was performed on the white particles of the Mo_5_C_0.5_ HEA in Figure 4. It was found that the white particles were enriched with the Mo element, which were the carbides of Mo combined with the C element. This was mainly because the Mo atom content exceeded the solid solution limit of the Mo_5_C_0.5_ HEA. 

**Figure 5:**
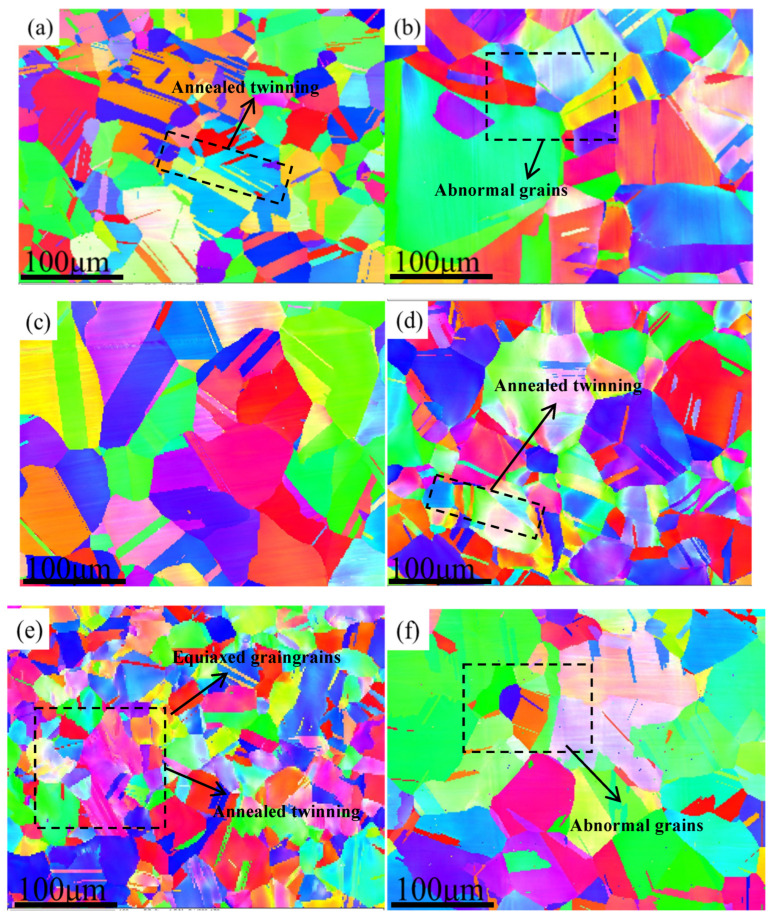
EBSD Image of HEAs: (**a**) Mo_3_, (**b**) Mo_4_, (**c**) Mo_5_, (**d**) Mo_3_C_0.5_, (**e**) Mo_4_C_0.5_, and (**f**) Mo_5_C_0.5_.

Figure 5 shows the EBSD image of hot-rolled HEAs. The alloy not only contained fully recrystallized grains, but it also contained deformed grains and a large amount of twin boundaries. Compared with as-cast alloys, the grain size was significantly smaller, and there was no component segregation or dendrite appearance. The alloy structure was composed of equiaxed grains and elongated grains, with a small amount of annealing twins. The uneven distribution of deformed grains could be observed in the grains. This was mainly attributed to the dynamic recrystallization process of the alloy during hot rolling, where the grains continuously recovered and grew during the deformation process, resulting in a decrease in the grain size [31], which helped to achieve fine grain strengthening and to improve the mechanical properties of the alloy. A similar carbon-doped Fe_40.4_Ni_11.3_Mn_34.8_A_l7.5_Cr_6_ alloy was studied by Wu [32]; the microstructure of equiaxed grains and elongated grains, as well as the dense dislocation walls, indicated that the alloy did not fully recrystallize, which was consistent with this result. As the Mo content increased without adding C, the grain size of the alloy increased from 17 μm to 47 μm. However, after adding 0.5 at.% carbon, the grain size of the alloy increased in the Mo_3_C_0.5_ and Mo_5_C_0.5_ HEAs, while that the grain size of Mo_4_C_0.5_ decreased significantly. 

**Figure 6:**
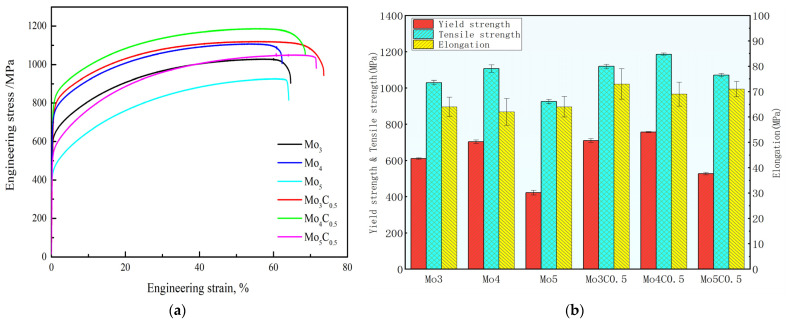
Tensile stress–strain curve of hot-rolled HEAs (**a**) and mechanical properties with error bars (**b**).

Figure 6 shows the tensile engineering stress–strain curve of the HEAs. It can be seen that, without the addition of carbon, the yield strength and tensile strength of the alloy showed a trend of first increasing and then decreasing with the increase of Mo content, while the elongation showed a trend of first decreasing and then increasing. The yield strength and tensile strength of the Mo_4_ alloy increased to 703 MPa and 1107 MPa, respectively(see Table 2). The reason for this is that the Mo element increased the lattice distortion, the lattice friction stress, and the solid solution strengthening effect. Due to the significant increase in the grain size, the strength of the Mo_5_ HEA decreased significantly. On the other hand, the precipitation phase of σ and μ gradually increased with the increase in Mo contents, which can be segregated at grain boundaries and can cause the early cracking of grain boundaries under a tensile test. After adding 0.5 at.% C content, the yield strength and tensile strength of the HEAs showed the same trend compared to the HEAs with no carbon added. However, the strength of C and Mo microalloyed HEAs increased by 99 MPa, 54 MPa, and 105 MPa, respectively, which was the result of interstitial solid solution strengthening caused by carbon atoms. From Figure 6b, it can be seen that the hot-rolled Mo_4_C_0.5_ HEA exhibited excellent comprehensive mechanical properties; its yield strength, tensile strength, and elongation were 757 MPa, 1186 MPa, and 69%, respectively. This was due to the elimination of structural segregation and the reduction in grain size in the Mo_4_C_0.5_ alloy after hot rolling. It is very significant to us in this research work. It is appropriate to infer that work hardening is caused by pinning the dislocations by the precipitates, such as carbides [33]. The strengthening mechanism is the combination of fine grain strengthening, interstitial solid solution strengthening, precipitation strengthening, and solid solution strengthening.

**Figure 7:**
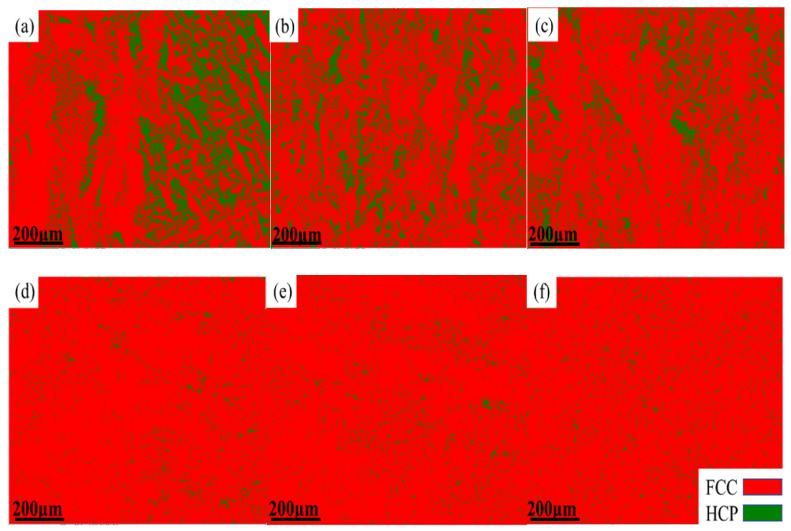
EBSD phase diagram of HEAs before deformation: (**a**) Mo_3_, (**b**) Mo_4_, (**c**) Mo_5_, (**d**) Mo_3_C_0.5_, (**e**) Mo_4_C_0.5_, and (**f**) Mo_5_C_0.5_.

**Figure 8:**
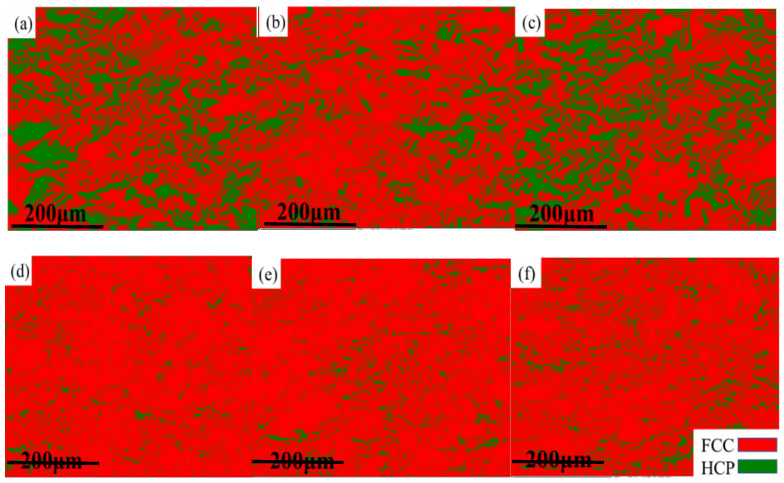
EBSD phase diagram of HEAs after deformation: (**a**) Mo_3_, (**b**) Mo_4_, (**c**) Mo_5_, (**d**) Mo_3_C_0.5_, (**e**) Mo_4_C_0.5_, and (**f**) Mo_5_C_0.5_.

**Figure 9:**
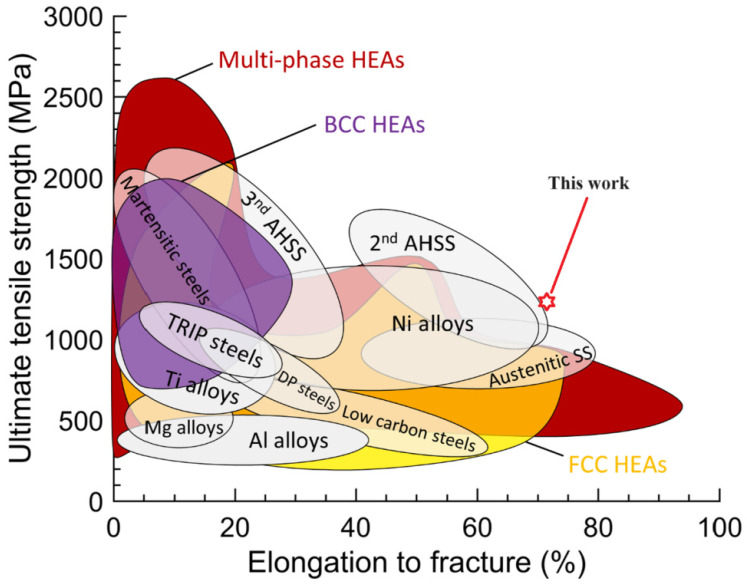
Ultimate tensile strength–ductility map of HEAs in the context of traditional alloys [34].

Figure 7 and Figure 8 show the EBSD phase diagrams of the HEAs before and after deformation. The red and green regions represent the FCC phase and HCP phase, respectively. It can be seen that, without the addition of carbon, the HCP phase significantly increased after deformation. This is because the alloy underwent a phase transformation-induced plasticity (TRIP) effect during tensile deformation, and some unstable FCC phases changed into HCP phases. After adding 0.5 at.% carbon, the alloy transformed into a phase with a large amount of FCC and a phase with a very small amount of HCP before deformation. This indicated that the addition of carbon element helped to enhance the stability of the FCC phase in the alloy, and the stacking fault energy of the alloy was relatively low at this time. After deformation, it could be observed that the HCP phase content increased, but the volume fraction of HCP phase decreased significantly compared to the HEAs without carbon addition. At this time, the deformation mechanism was no longer dominated by the TRIP effect but mainly by the twinning-induced plasticity (TWIP) effect. This is because the addition of a small amount of carbon element increased the stacking fault energy and the stability of the FCC phase of HEAs, which reduced the TRIP effect. The number of deformation twins at 60° increased significantly after deformation. Deformation twins were mainly generated in grains inclined towards <101> and <111> orientations, which indicated that the formation of deformation twins was closely related to the initial orientation of the grains. The relationship between grain orientation and deformation twinning is very similar to the trend of TWIP steel. In the process of deformation, dislocation plugs up on the twin interface, and the annealing twin boundary will gradually change into a small-angle grain boundary of less than 5°. The deformation mechanism was controlled simultaneously by dislocation slip and deformation twins. When the deformation was larger, the deformation twins occupied the positions of annealing twins and inhibited the dislocation movement, which caused the number of interfaces with the mismatched angle between 2° and 10° to increase. Deformation twins were mainly formed at grain boundaries of <111> and <100> grain orientations and mainly expanded along the <111>//TA orientation (//TA is defined as parallel to the direction of the tension axis). The TWIP effect made the twin the leading deformation mechanism. The dislocation slip and twinning mechanism competed with each other in the process of tensile deformation, which jointly affected the microstructure evolution. The generation of deformation twins hindered the dislocation slip and formed a dislocation accumulation zone, which ensued the uniform deformation and effectively delayed the occurrence of necking, finally causing excellent comprehensive mechanical properties for the Mo_4_C_0.5_ HEA. Apparently, the studied Mo_4_C_0.5_ HEA in this present work possesses excellent comprehensive mechanical properties, making it superior to a majority of reported HEAs and conventional metal alloys (see Figure 9). 

## 4. Conclusions

(1) There were FCC + HCP dual phases in Mo_3_, Mo_4_, and Mo_5_ HEAs when no C atoms were added. As the Mo content increased, the grain size of the alloy increased from 17 μm to 47 μm. However, only the FCC phase appeared after adding 0.5 at.% carbon, and the grain size of the Mo_4_C_0.5_ HEA decreased significantly.
(2) Due to the Mo atom content exceeding the solid solution limit, the carbides of Mo combined with C element appeared in the Mo_5_C_0.5_ HEA. The strength of C and Mo microalloyed HEAs had a significant increase compared to HEAs with no C added, which was the result of interstitial solid solution strengthening caused by carbon atoms.
(3) The Mo_4_C_0.5_ HEA exhibited excellent comprehensive mechanical properties, making it superior to a majority of reported HEAs and conventional metal alloys. Its yield strength, tensile strength, and elongation were 757 MPa, 1186 MPa, and 69%, respectively. The strengthening mechanism was the combination of fine grain strengthening, the TWIP effect, and solid solution strengthening.

## Figures and Tables

**Table 1:** Chemical composition of HEAs (at.%).

HEAs	Co	Cr	Fe	Ni	Mo	C
Mo_3_	40.000	25.000	16.000	16.000	3.000	0.000
Mo_4_	40.000	25.000	15.500	15.500	4.000	0.000
Mo_5_	40.000	25.000	15.000	15.000	5.000	0.000
Mo_3_C_0_._5_	39.800	24.875	15.920	15.920	2.9850	0.500
Mo_4_C_0_._5_	39.800	24.875	15.4225	15.4225	3.9800	0.500
Mo_5_C_0.5_	39.800	24.875	14.9250	14.9250	4.9750	0.500

**Table 2:** Mechanical properties of HEAs.

HEAs	Yield Strength (MPa)	Tensile Strength (MPa)	Elongation (%)
Mo_3_	611	1030	64
Mo_4_	703	1107	62
Mo_5_	422	925	64
Mo_3_C_0.5_	710	1120	73
Mo_4_C_0.5_	757	1186	69
Mo_5_C_0.5_	527	1071	71

## Data Availability

Data will be made available upon request.
